# Supplementary material for: Deletion of wfs1 Impairs Oligodendrocyte Precursor Cells Dorsal Distribution and Myelination Through the wfs1-hmgcs1 Axis in Zebrafish
Source: Biology (Basel). 2026 Aug 16;15(16):1402. doi: 10.3390/biology15161402 (PMC13510151; doi:10.3390/biology15161402)
Supplement: Supplementary file 1 [file biology-15-01402-s001.zip › File S1-Transcriptome sequencing data and differentially expressed genes (DEGs) in wfs1-- zebrafish at 5 dpf/2.KEGG/wfs1KOvsWT/DOWN/src/dre04020.html]

dre04020


- dre:368717

- Down regulated genes

368717\_1(-1.74128930765284)

- dre:565241

- Down regulated genes

565241\_2(-6.09874532631292)

- dre:497384

- Down regulated genes

497384\_1(-2.05267237809407)

- dre:544664

- Down regulated genes

544664(-5.14896748950436)
- dre:386856

- Down regulated genes

386856\_1(-1.99487147218035) novel.946(-6.7676180476835)
- dre:569566

- Down regulated genes

569566(-1.00953785382545)
- dre:571431

- Down regulated genes

571431(-6.42862549702529)
- dre:796537

- Down regulated genes

796537\_1(-2.50164719187441)

- dre:387296

- Down regulated genes

387296\_1(-5.24544706670971)
- dre:58147

- Down regulated genes

58147\_1(-3.00265513562539)
- dre:259258

- Down regulated genes

259258(-3.56445279283737)
- dre:570493

- Down regulated genes

570493\_1(-1.68806351340199)

- dre:566671

- Down regulated genes

566671(-1.07930365706202)

- dre:492486

- Down regulated genes

492486\_1(-1.65362606223537)
- dre:100000981

- Down regulated genes

100000981\_2(-1.61456337824489)
- dre:100137117

- Down regulated genes

100137117(-1.33851850264817)

- dre:353247

- Down regulated genes

353247(-1.16019504230133)

- dre:100005590

- Down regulated genes

100005590\_1(-4.43307927362236)

- dre:100005049

- Down regulated genes

100005049(-1.30748784788409)
- dre:560564

- Down regulated genes

560564\_2(-4.02056550705436)
- dre:103909601

- Down regulated genes

378480(-1.33145438810737)

Close
